# Altered maternal profiles in corticotropin-releasing factor receptor 1 deficient mice

**DOI:** 10.1186/1471-2202-8-17

**Published:** 2007-03-01

**Authors:** Stephen C Gammie, Emily D Bethea, Sharon A Stevenson

**Affiliations:** 1 Department of Zoology, University of Wisconsin, Madison, Wisconsin, 53706, USA; 2 Neuroscience Training Program, University of Wisconsin, Madison, Wisconsin, 53706, USA

## Abstract

**Background:**

During lactation, the CNS is less responsive to the anxiogenic neuropeptide, corticotropin-releasing factor (CRF). Further, central injections of CRF inhibit maternal aggression and some maternal behaviors, suggesting decreased CRF neurotransmission during lactation supports maternal behaviors. In this study, we examined the maternal profile of mice missing the CRF receptor 1 (CRFR1). Offspring of knockout (CRFR1-/-) mice were heterozygote to offset possible deleterious effects of low maternal glucocorticoids on pup survival and all mice contained a mixed 50:50 inbred/outbred background to improve overall maternal profiles and fecundity.

**Results:**

Relative to littermate wild-type (WT) controls, CRFR1-/- mice exhibited significant deficits in total time nursing, including high arched-back, on each test day. Consistent with decreased nursing, pups of CRFR1-deficient dams weighed significantly less than WT offspring. Licking and grooming of pups was significantly higher in WT mice on postpartum Day 2 and when both test days were averaged, but not on Day 3. Time off nest was higher for CRFR1-/- mice on Day 2, but not on Day 3 or when test days were averaged. Licking and grooming of pups did not differ on Day 2 when this measure was examined as a proportion of time on nest. CRFR1-/- mice showed significantly higher nest building on Day 3 and when tests were averaged. Mean pup number was almost identical between groups and no pup mortality occurred. Maternal aggression was consistently lower in CRFR1-/- mice and in some measures these differences approached, but did not reach significance. Because of high variance, general aggression results are viewed as preliminary. In terms of sites of attacks on intruders, CRFR1-/- mice exhibited significantly fewer attacks to the belly of the intruder on Day 5 and when tests were averaged. Performance on the elevated plus maze was similar between genotypes. Egr-1 expression differences in medial preoptic nucleus and c-Fos expression differences in bed nucleus of stria terminalis between genotype suggest possible sites where loss of gene alters behavioral output.

**Conclusion:**

Taken together, the results suggest that the presence of an intact CRFR1 receptor supports some aspects of nurturing behavior.

## Background

Successful rearing of offspring in rodents involves the expression of a number of maternal behaviors including nursing, pup retrieval, nest building, and defense of offspring (maternal aggression) [1]. In association with lactation, the CNS becomes less sensitive to the anxiogenic neuropeptide, corticotropin-releasing factor (CRF) [2,3], suggesting that decreased CRF neurotransmission during lactation could support maternal care. In support of this idea, recent work has shown that central injections of CRF and related peptides dose-dependently impair maternal aggression in mice [4,5]. Also, an earlier study found centrally injected CRF decreased maternal care in maternally sensitized virgin female rats [6]. Recent quantitative trait loci (QTL) analysis identified 23 possible QTLs in mice as being associated with quality of maternal care [7]. Among possible gene candidates in those were both CRF and CRF receptor 1 (CRFR1). Although accumulating evidence links CRF and it primary receptor to maternal care, no study to date has examined in detail how loss of the CRFR1 gene affects maternal responding and aggression.

CRF triggers peripheral increases in stress hormones (glucocorticoids) [8] as well as behavioral responses to stress (fear and anxiety) by acting within the CNS (for reviews, see [9,10]). CRF acts primarily on CRFR1 [11-13], but can also activate CRF receptor 2 (CRFR2) with less efficacy [14,15]. We recently showed that mice missing CRFR2 exhibit impaired maternal aggression, but normal pup retrieval behavior [16] and speculated that the overproduction of CRF in these CRFR2 knockout mice acting on an intact CRFR1 was responsible for the deficits in aggression.

A valuable approach for understanding the role of a gene in behavior is to examine behavioral changes when that gene is removed. CRFR1-/- mice have been reported previously to display elevated levels of CRF in the paraventricular nucleus, but not in amydala or other regions and to show decreased indices of fear and anxiety [12]. Levels of CRFR2 in these mice was not altered. CRFR1-/- mice showed normal growth and reproduction, but CRFR1-/- pups of CRFR1-/- dams died within a few days of birth due to lung dysplasia likely due to a combination of low levels of glucocorticoids in the CRFR1-/- dams and pups [12]. Knockout pups of CRF mutant mice show a more severe form of lung dysplasia and die within hours of birth due to low glucocorticoids in both dams and pups [17]. Importantly, heterozygote offspring of CRF mutant mothers show normal glucocorticoid levels and thus are resistant to maternal corticosterone deficiencies [17].

The aim of this study was to examine whether or how loss of CRFR1 affected a range of maternal behaviors, including maternal defense. Because inbred strains show overall decreased fecundity in terms of litter size and reproduction rate relative to outbred mice, we examined the loss of the CRFR1 gene in a mixed inbred/outbred background. Further, to overcome any deleterious effects of maternal deficiencies in glucocorticoids in CRFR1-/- mice, all mice were mated with outbred mice so that progeny would not be missing the CRFR1 gene. Given that centrally injected CRF impairs maternal aggression and some behaviors and that the lactating CNS is less responsive to CRF, we speculated that CRFR1-/- mice would show normal or even heightened levels of some maternal behaviors and aggression. However, if some levels of CRF acting on CRFR1 are necessary for maternal responding, it would be expected that certain behaviors would be impaired by the loss of the CRFR1 gene. To gain insights into how CRFR1 contributes to maternal care, we also examined c-Fos and egr-1 expression in untested, naturally behaving WT and CRFR1-/- mice.

## Results

### Maternal behaviors

**Figure 1:**
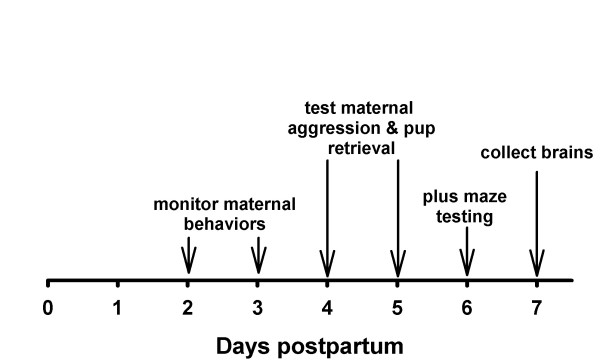
Overview of the timeline for behavioral observations and testing. Birth of pups is considered postpartum Day 0.

**Figure 2:**
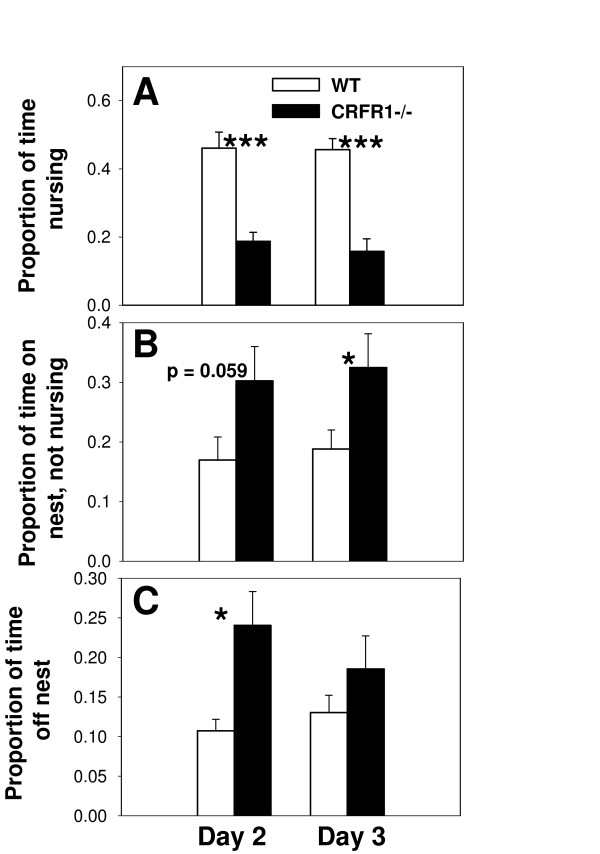
Analysis of nursing related behaviors in WT and CRFR1-/- mice. On postpartum Days 2 and 3, CRFR1-/- females exhibited significant deficits in proportion of time nursing relative to WT mice (A). Further, even while on the nest, CRFR1-/- mice spent less proportion of time nursing pups relative to WT mice in Day 3 (B). CRFR1-/- mice spent significantly more time away from the nest on postpartum Day 2 relative to WT mice, but differences between groups on Day 3 did not reach significance (C). Bars represent means ± SE. White bars indicate WT mice and black bars indicate CRFR1-/- mice. * = p < 0.05; *** = p < 0.001; one-way ANOVA (A) and (B) and one-way ANOVA on ranks for (C).

Maternal behaviors were examined on postpartum Days 2 and 3 and a timeline of all testing is shown in Fig. 1. On postpartum Day 2, WT mice spent a significantly higher proportion of time nursing (all nursing combined) than CRFR1-/- mice (F(1,24) = 19.08; p < 0.001; one-way ANOVA) (Fig. 2A). Further, while on the nest, CRFR1-/- mice showed a non-significant trend towards a lower proportion of time nursing (F(1,24) = 3.93; p = 0.059; one-way ANOVA (Fig. 2B) and spent a significantly greater proportion of time away from the nest (Q(1,24) = 2.71; p < 0.05; one-way ANOVA). Consistent with results of Day 2, on postpartum Day 3, WT mice spent a significantly higher proportion of time nursing than CRFR1-/- mice (F(1,24) = 35.00; p < 0.001; one-way ANOVA) (Fig. 2A), while CRFR1-/- mice spent a significantly lower proportion of time nursing while on nest (F(1,24) = 5.12; p < 0.05; one-way ANOVA) (Fig. 2B). Proportion of time away from the nest did not differ (F(1,24) = 1.59; p = 0.219; one-way ANOVA) (Fig. 2C). When examined as a proportion of time on nest, proportion of time nursing (all forms combined and individually) was significantly higher in WT relative to CRFR1-/- mice on both test days (data not shown). When the mean result for each mouse over the two test days was examined, proportion of time nursing still differed significantly between groups (p < 0.001, ANOVA on Ranks), including the different forms (data not shown). However, proportion of time off nest was just above significance (p = 0.052, one-way ANOVA).

**Figure 3:**
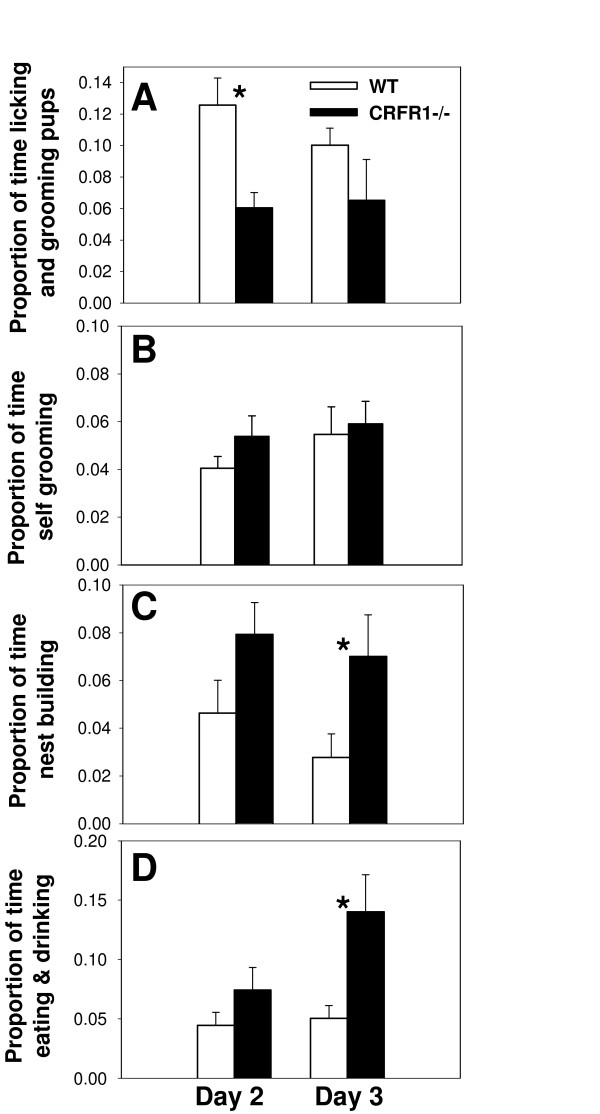
Analysis of maternal and non-maternal behaviors in WT and CRFR1-/- mice. CRFR1-/- mice spent a significantly lower proportion of time licking and grooming offspring postpartum Day 2 relative to WT mice, but differences between groups on Day 3 did not reach significance (A). Self grooming behavior did not differ between genotype on either test day (B). CRFR1-/- mice spent a higher proportion of time nest building than WT mice and these differences reached significance on postpartum Day 3 (C). CRFR1-/- mice spent a higher proportion of time eating and drinking than WT mice and these differences reached significance on postpartum Day 3 (D). Bars represent means ± SE. White bars indicate WT mice and black bars indicate CRFR1-/- mice. * = p < 0.05; ANOVA on ranks for (A), (C), and (D).

On postpartum Day 2, WT mice spent significantly higher proportion of time licking and grooming pups than CRFR1-/- mice (Q(1,24) = 2.35; p = 0.018; ANOVA on ranks) (Fig. 3A), whereas self grooming did not differ between genotype (F(1,24) = 2.06; p = 0.164; one-way ANOVA (Fig. 3B). However, as a proportion of time while on nest, licking and grooming of pups did not differ between groups (p = 0.164). No differences in proportion of time nest building (F(1,24) = 2.69; p = 0.115; one-way ANOVA (Fig. 3C) and eating and drinking (F(1,24) = 2.08; p = 0.162; one-way ANOVA (Fig. 3D) were found. On postpartum Day 3, proportion of time licking and grooming pups did not differ between genotype (F(1,24) = 1.3; p = 0.164; one-way ANOVA (Fig. 3A). Self grooming again did not differ between genotype on Day 3 (F(1,24) = 0.0; p = 0.790; one-way ANOVA (Fig. 3B). CRFR1-/- mice spent a significantly higher proportion of time nest building (Q(1,24) = 2.55; p < 0.05; ANOVA on Ranks, Dunn's Method) (Fig. 3C) and eating and drinking (Q(1,24) = 2.05; p < 0.05; ANOVA on Ranks, Dunn's Method (Fig. 3D). When the mean result for each mouse over the two test days was examined, licking and grooming of pups was significantly higher in WT mice (p = 0.007, one way ANOVA) and nest building was significantly lower in WT mice (p = 0.011, one-way ANOVA). Self grooming and eating and drinking did not differ between groups (data not shown).

**Figure 4:**
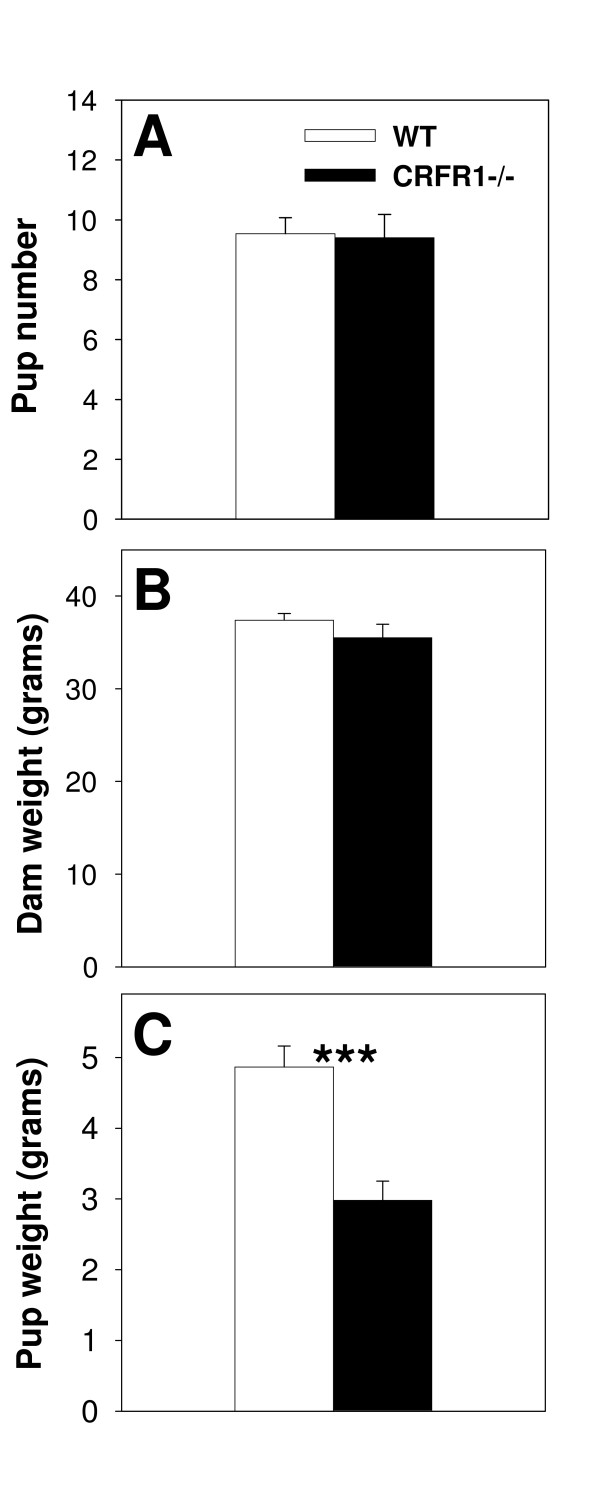
Profile of WT and CRFR1-/- dams and pups. Neither pup number (A) nor dam weight (B) differed between genotype. In contrast, mean pup weight on postpartum Day 6 was significantly higher in WT relative to CRFR1-/- mice (C). Bars represent means ± SE. White bars indicate WT mice and black bars indicate CRFR1-/- mice. *** = p < 0.001; one-way ANOVA.

Mean pup number was almost identical between genotypes and did not differ significantly (F(1,24) = 0.01; p = 0.885; one-way ANOVA) (Fig. 4A). Although pup number did not differ between genotypes, it was used as a covariate for analyzing some aggressive measures (see below). In terms of mean dam weight (in grams) on postpartum Day 6, no differences existed between genotypes (F(1,24) = 1.64; p = 0.212; one-way ANOVA) (Fig. 4B). In contrast to dam weight and litter size, the weight of individual pups and total pup mass on postpartum Day 6 differed significantly between genotypes. Mean total weight of pups (in grams) was significantly higher in WT (44.7 ± 2.0) relative to CRFR1-/- mice (27.7 ± 3.2) (F(1,24) = 21.8; p < 0.001; one-way ANOVA). Further, mean weight of individual pups was significantly higher in WT relative to CRFR1-/- mice (F(1,24) = 19.1; p < 0.001; one-way ANOVA) (Fig. 4C). Proportion of time nursing on Day 2 and 3 was significantly correlated with Day 6 pup weight when all animals were examined together (Day 2, correlation coefficient = 0.557, p = 0.003) and (Day 3, correlation coefficient = 0.661, p = 0.0003).

No differences were found between groups in terms of latency from pairing to birth (21.6 ± .03 days for WT; 22.5 ± 0.4 days for CRFR1-/-; p = 0.114).

### Maternal aggression

**Figure 5:**
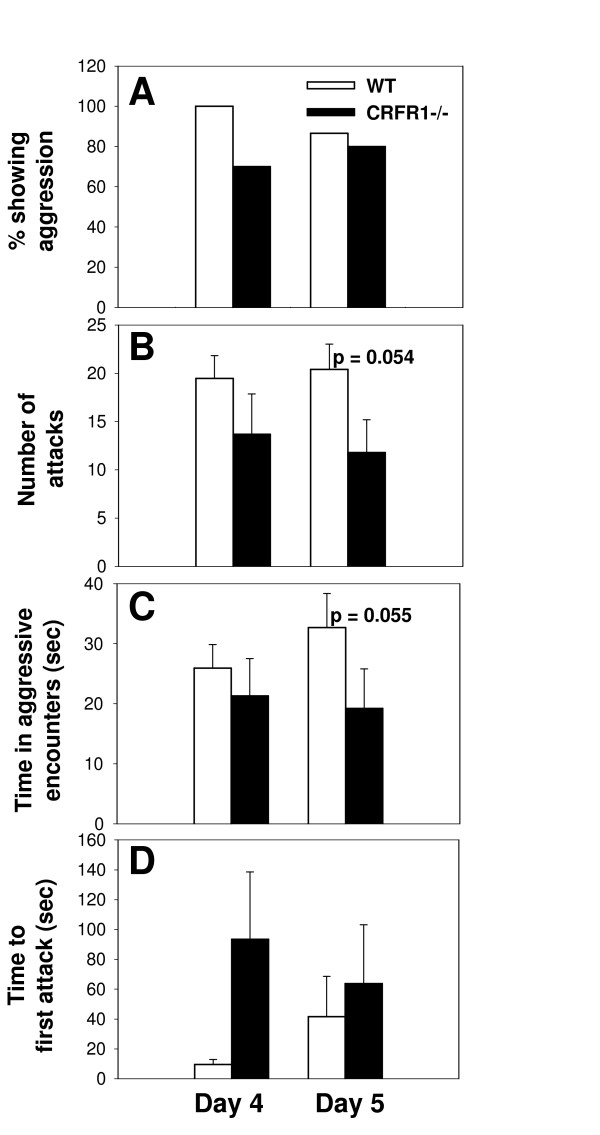
Analysis of maternal aggression in WT and CRFR1-/- mice. Using a resident-intruder paradigm, CRFR1-/- mice show decreased aggression relative to WT mice, but these difference do not reach significance in terms of the percentage of females showing any aggression (A), the average number of attacks (B), the average amount of time in aggressive encounters (C), and the average latency to first attack (D) when examined on either postpartum Day 4 or 5. Bars represent means ± SE. White bars indicate WT mice and black bars indicate CRFR1-/- mice. Data that were non-normally distributed were examined via non-parametric tests (see Methods and Results for more details). When a possible outlier from the CRFR1-/- was removed, significant differences in aggression were found for some measures (see Results for details).

Maternal aggression was consistently lower in CRFR1-/- mice, but these differences approached, but did not reach significance using most statistical approaches. For example, maternal aggression did not differ between CRFR1-/- and WT mice on postpartum Day 4 in terms of percentage showing aggression (Q(1,24) = 3.75; p > 0.05; ANOVA on Ranks, Dunn's Method) (Fig. 5A), number of attacks (F(1,24) = 1.69; p = 0.206; one-way ANOVA (Fig. 5B), time in aggressive encounters (F(1,14) = 0.44; p = 0.511; one-way ANOVA (Fig. 5C), or time to first attack (H(1,24) = 0.45; p = 0.501; ANOVA on Ranks (Fig. 5D). On postpartum Day 5, in terms of number of attacks, CRFR1-/- mice exhibited fewer attacks, but these differences were just above significance (F(1,24) = 4.11; p = 0.054; one-way ANOVA (Fig. 5B). In terms of total time aggressive on Day 5, aggression differences (lower in CRFR1-/-) were also found to be just above significance using a non-parametric test (H(1,24) = 3.68; p = 0.055; ANOVA on Ranks (Fig. 5B). When data were transformed to achieve normality using a power 0.7 transform, neither a one-way ANOVA (F(1,24) = 2.64; p = 0.117) nor an ANCOVA incorporating pup number as a covariate (F(1,24) = 3.17; p = 0.088) indicated a difference between groups. When the mean result for each mouse over the two test days was examined, no differences were found between groups in terms of either time to first attack (p = 0.358, ANOVA on Ranks), number of attack (p = 0.087, one-way ANOVA), or total time aggressive (p = 0.075, ANOVA on Ranks).

When using a criterion of removing outliers more than 2 standard deviations from the mean, one knockout mouse can be excluded. With this data set, significant differences between groups were found in terms of number of attacks on Days 4 and 5 (p < 0.05 each day) and time aggressive on Day 5 (p < 0.05).

In terms of the breakdown of total agonistic behavior (including clawing and lunging), WT mice exhibited a significantly greater percentage attacks to the ventral portion of the mid-section (including belly) relative to CRFR1-/- mice on Day 5 (H(1,24) = 5.82; p < 0.05; ANOVA on Ranks). For all other sites of attack, no differences existed between genotype (data not shown). On average for Day 4, WT and CRFR1-/- mice exhibited, respectively, 7 and 4% of attacks to the ventral portion of the mid-section (including belly), 50 and 54% of attacks to the back/flank region, 28 and 35% of attacks to the head/neck region, and 15 and 7% clawing or lunging towards the intruder. On average for Day 5, WT and CRFR1-/- mice exhibited, respectively, 13 and 2% of attacks to the ventral portion of the mid-section (including belly), 60 and 44% of attacks to the head/neck region, 20 and 39% of attacks to the head/neck region, and 7 and 15% clawing or lunging towards the intruder. When results from both test days were combined, attacks to the belly were significantly higher in WT mice (p = 0.028, ANOVA on Ranks), but other sites of attack did not differ between groups (data not shown).

### Pup retrieval

No significant differences in pup retrieval were observed between WT and CRFR1-/- mice on either Day 4 (H(1,24) = 1.12; p = 0.290; one-way ANOVA on ranks; 1^st ^pup; H(1,24) = 1.21; p = 0.270; one-way ANOVA on ranks; 4^th ^pup) or Day 5 (F(1,24) = 0.01; p = 0.889; one-way ANOVA; 1^st ^pup; H(1,17) = 0.00; p = 0.976; one-way ANOVA on ranks; 4^th ^pup). On average, the retrieval time (in sec) for 1^st ^pup was 17 for WT and 32 for CRFR1-/- mice on Day 4 and 28 for WT and 25 for CRFR1-/- mice on Day 5. On average, the retrieval time (in sec) for 4^th ^pup was 89 for WT and 71 for CRFR1-/- mice on Day 4 and 92 for WT and 93 for CRFR1-/- mice on Day 5.

### Elevated plus maze test

On postpartum Day 6, total time in open arms (in sec) (WT = 13.8 ± 5.2; CRFR1-/- = 16.9 ± 8.4) (H(1,24) = 0.16; p = 0.681; ANOVA on Ranks) and closed arm (in sec) (WT = 205.6 ± 17.1; CRFR1-/- = 208.4 ± 11.7) (F(1,24) = 0.01; p = 0.907; ANOVA) did not differ between genotypes. Further, for no other measure, including latency to open arm (in sec) (WT = 174.8 ± 34.9; CRFR1-/- = 205.9 ± 42.9), number of visits to open arms (WT = 1.7 ± 0.6; CRFR1-/- = 1.2 ± 0.7), number of visits to closed arms (WT = 10.0 ± 1.6; CRFR1-/- = 10.1 ± 0.8), number of visits to middle square (WT = 9.9 ± 1.2; CRFR1-/- = 11.7 ± 1.2), and time in middle (WT = 79.3 ± 14.8; CRFR1-/- = 74.4 ± 8.7), were differences detected between genotype.

### c-Fos and Egr-1 immunoreactivity in WT and CRFR1-/- dams

**Figure 6:**
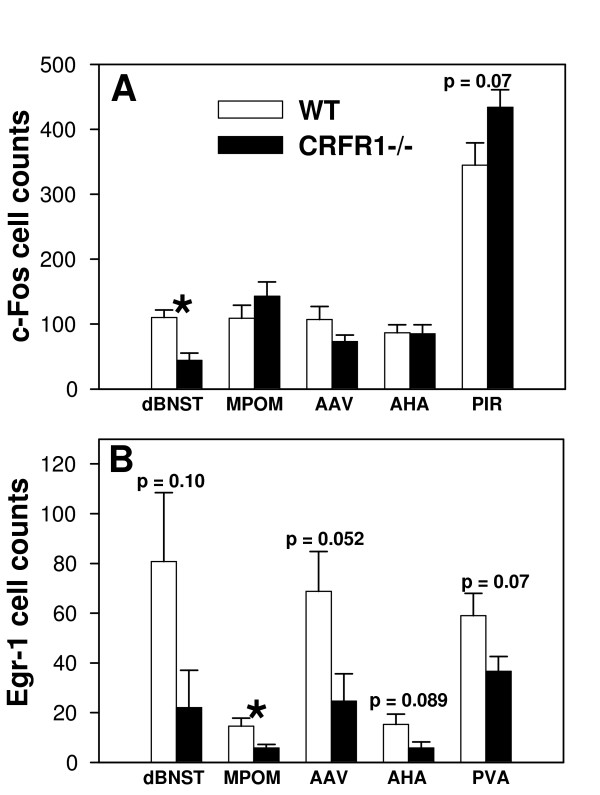
c-Fos and Egr-1 immunoreactivity in WT and CRFR1-/- mice. In terms of c-Fos immunoreactivity (A), significantly lowers levels of cell counts were found in BNSTd in CRFR1-/- relative to WT mice. In terms of Egr-1 immunoreactivity (B), significantly lower levels of cell counts were found in MPOM in CRFR1-/- relative to WT mice. Bars represent means ± SE. White bars indicate WT mice and black bars indicate CRFR1-/- mice. * = p < 0.05; one-way ANOVA. Other abbreviations: AAV = anterior amygdaloid area; AHA = anterior hypothalamic area; PVA = paraventricular nucleus of the thalamus.

Baseline neuronal activity in WT and CRFR1-/- mice was examined on postpartum Day 7. In terms of c-Fos immunoreactivity, differences between groups were only found in bed nucleus of stria terminalis dorsal (BNSTd) (F(1,8) = 16.0; p = 0.005; one-way ANOVA) (Fig. 6A). A higher level of c-Fos was found in piriform cortex (PIR) in CRFR1-/- mice, but these differences did not reach significance (F(1,28) = 4.26; p = 0.078; one-way ANOVA) (Fig. 6A).

**Figure 7:**
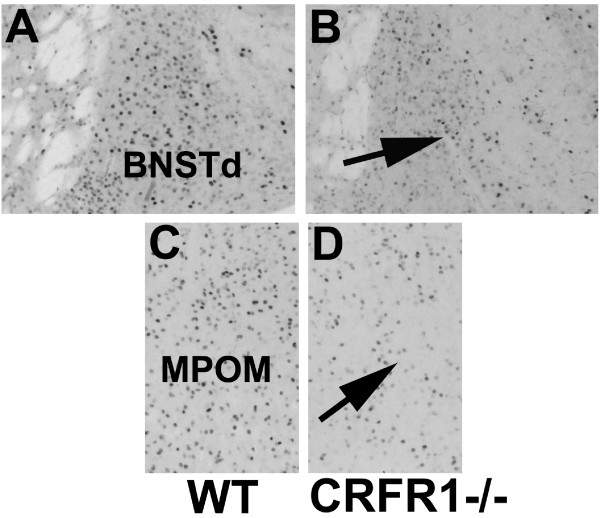
Examples of c-Fos immunoreactivity in BNSTd for WT (A) and CRFR1-/- (B) mice. Examples of Egr-1 immunoreactivity in MPOM in WT (C) and CRFR1-/- (D) mice.

In terms of Egr-1 immunoreactivity, differences between groups were only found in medial preoptic nucleus (MPOM) (F(1,8) = 6.19; p = 0.038; one-way ANOVA) (Fig. 6B). Consistent with c-Fos increases, heightened Egr-1 immunoreactivity was found in BNSTd in WT mice, but these differences did not reach significance (F(1,28) = 3.46; p = 0.100; one-way ANOVA) (Fig. 6B). Additional regions that approached significance between genotypes are shown in Fig. 6B. Examples of c-Fos and Egr-1 immunoreactivity are shown in Fig. 7.

## Discussion

In the present study, we show that knockout mice missing CRFR1 exhibit deficiencies in nurturing behavior that include decreases in nursing and licking and grooming of pups. Interestingly, pup retrieval is not altered in CRFR1-/- mice and nest building is enhanced. Maternal aggression was lower in knockout mice, but these differences did not reach significance using most tests. High variance in the aggression results makes these findings preliminary. Because the loss of CRFR1 alters both central signaling and glucocorticoid production, this study provides a detailed maternal profile of the CRFR1-/- mice, but does not examine where or how the phenotype can be rescued. Future studies using glucocorticoid replacement, cross fostering of pups, and spatial and temporal inactivation of CRFR1 activity will be required to elucidate the basis of the maternal alterations.

Increasing central CRF levels (via icv injections) (which would presumably increase activation of CRFR1) decreases maternal responding in maternally sensitized virgin rats [6]. Further, studies in humans indicate that dysregulation of CRF neurotransmission is linked to some forms of depression [18] and depression, itself, has been linked to decreases in maternal care [19]. The simplest interpretation of our finding that loss of CRFR1 impairs certain aspects of maternal care (nursing and licking and grooming of pups) is that a certain amount of CRF tone acting on CRFR1 is required for full maternal behavior expression. Hence, with tone too low (loss of CRFR1 gene) or too high, maternal care is impaired. Thus, CRF acting on CRFR1 could have an inverted U-shaped effect on maternal care. A link between CRFR1 and maternal care was also recently suggested by a QTL study in mice [7]. Because CRF and its related peptides, Ucn 1 and 3, can activate CRFR2 [14,20-22], it is possible that increased activation of CRFR2 compensates for the loss of CRFR1 and contributes to alterations in maternal care. The differences in licking and grooming of pups is not as robust as for nursing and indeed, on Day 2, licking and grooming as a proportion of time on nest (CRFR1-/- mice spent less time on nest) does not differ between groups. However, mean licking and grooming of pups does differ significantly between groups whereas time off nest does not (although this is just above significance). The findings of altered licking and grooming of pups should be interpreted more cautiously given that altered time on nest could contribute to the phenotype. The elevation of nest building in CRFR1-/- mice suggests that the deletion of this gene does not uniformly adversely affect maternal care and may enhance some forms of it. An examination of maternal behaviors in double knockouts of both receptors would help determine whether or how the receptors might work together to regulate maternal responding.

A drawback of knock-out studies is that the deletion of a gene may have developmental or compensatory effect that is separable from the functional use of the protein product as adults [23]. Also, inbred mice are used as a background for most knockouts in mice, but inbred mice tend to have decreased fecundity relative to outbred mice. Because we were interested in observing maternal care in CRFR1-deficient mice, we took additional steps to improve overall levels of reproduction and allow better levels of maternal care against which to examine loss of the gene. Outbred hsd:ICR mice exhibit high fecundity and produce on average 12 pups per litter, almost twice the size of most inbred strains, including C57BL/6, the predominant background strain for most knockout mice. We have recently used the hsd:ICR (also known as CD-1) strain to select for high levels of maternal aggression [24]. By crossing inbred mice with a deficiency into hsd:ICR mice selectively bred for high maternal aggression, all mice in this study had a mixed inbred:outbred (50:50) background that produced high fecundity in both WT and CRFR1-/- groups as evidenced by pregnancy rate and litter size. By using outbred breeder males we ensured that offspring of CRFR1-/- mice would be heterozygote and thus genotype of offspring was less likely to influence dam behavior. Because the genome of inbred mice has been reduce to single alleles, genetic interactions are decreased and it has been suggested that examinations of missing genes on a more variable, outbred background may be more relevant to understanding the role of genes in humans [25]. Given that genetic background can affect behavioral phenotype in knockout mice [25], it will be valuable in future studies to examine aggressive responding in CRFR1 mutant mice with different genetic backgrounds (including inbred and outbred strains).

CRFR1-/- mice have previously been shown to exhibit lower than normal levels of glucocorticoids relative to WT mice, but a significant increase in corticosterone in response to a stressor occurs in CRFR1-/- mice [12]. The greatest difference in glucocorticoid levels in WT and CRFR1-/- females occurs in late afternoon (after behavioral testing in this study) when WT females exhibit a surge, but CRFR1-/- females do not. Given differences in corticosterone between genotype, altered levels of this steroid could alter gene expression in the CNS that interacts with a CRFR1-deficient CNS in a complex manner to affect behavior. Recent work indicates that glucocorticoids in rats support certain maternal behaviors, such as licking and grooming of pups and increasing time in nest, but not nursing *per se *[26]. Further, more recent work implicates glucocorticoids in maternal memory that would facilitate maternal care [27]. Although differences in glucocorticoid levels could contribute to some aspects of the behavioral differences in genotype, corticosterone replacement was unable to rescue anxiety measure differences between genotypes [12], suggesting some aspects of behavioral profiles in CRFR1-/- mice are not glucocorticoid-dependent. Future studies can address this issue by regulating corticosterone levels among the groups. An additional approach to understand the specificity of CRFR1 in maternal behavior would be to use site-directed injections of a CRFR1 antagonist and then examine behavioral responding.

CRFR1-/- pups of CRFR1-/- dams die within a few days of birth due to lung dysplasia due to low levels glucocorticoids in the CRFR1-/- dams [12]. This profile is similar for knockout pups of CRF mutant mice that show a more severe form of lung dysplasia and die within hours of birth due to low maternal glucocorticoids [17]. CRFR1-/- mice treated with glucocorticoids during pregnancy and early lactation prevents pup death [12], but detailed maternal care and offspring trajectories were not reported for this treatment. Importantly, it has been shown that heterozygote offspring of CRF mutant mothers show normal glucocorticoid levels and thus are resistant to maternal corticosterone deficiencies [17]. In this study, all offspring of CRFR1-/- mice were heterozygote and should have been producing normal levels of glucocorticoids. The lack of any pup death in CRFR1-/- mice supports the rescue effect of having one intact copy of CRFR1 in offspring. Having both genotypes foster pups derived from a different group of mice in future work would eliminate any role that pup genotype or exposure to maternal environment could have on maternal behavior.

The association between decreased nursing by CRFR1-/- dams and decreased pup weight is striking and suggests the deficits lie in the dam and not the offspring. CRFR1-/- dams showed elevated eating and drinking, time off nest, and nest building relative to WT mice, indicating the CRFR1-/- dams did not suffer from an overall decrease in activity, but rather had a shift in behavioral profiles. Further, CRFR1-/- dams nursed significantly less even if one just examines percentage of total time on nest, which suggests a lack of propensity in CRFR1-/- mice to nurse even with stimuli immediately present. The lack of difference between genotype in pup retrieval suggests the loss of the CRFR1 gene could specifically affect more passive rather than active behaviors. As indicated above and below, the results on maternal aggression are considered preliminary. However, decreases in high arched-back nursing (which involves maintaining a kyphotic posture) was also decreased in CRFR1-/- mice. The highly significant correlation between proportion of time nursing and pup weight across all mice suggests our observations across two days were sufficient to track behavioral differences that could provide a concrete explanation for differences in pup weight. Because we did not monitor maternal behaviors at multiple times during the day and night, we cannot rule out the possibility that alterations in circadian cycle between genotypes explains our nursing differences. However, to date no such differences in circadian rhythm have been reported between these genotypes.

Previous findings have indicated that CRF neurotransmission is decreased in association with lactation. In support of this, during lactation in rodents (1) the CNS is less responsive to centrally injected CRF [2], (2) the CNS shows decreased response to stressors [28], (3) CRF-enhanced startle response is decreased [3], and (4) females exhibit decreased fear and anxiety using a wide range of testing paradigms (for review, see [29], but also see [30]). It has previously been speculated that decreased fear and anxiety (possibly via decreased CRF neurotransmission) during lactation may support maternal aggression by increasing the likelihood that a dam will attack a normally fear-evoking stimulus [4,29]. In support of this idea, we recently have shown that central injection of CRF dose-dependently inhibits maternal aggression in mice [4]. In some cases, stressors applied pre-partum (which could elevate central CRF release) impair maternal defense [31,32] and CRF neurotransmission may be decreased during lactation to help prevent environmental stressors (acting via CRF) from unduly impairing the defense of offspring.

Aggression levels were consistently lower in knockout mice and the high variance could have confounded results. As indicated, the differences in aggression between groups approached significance in a number of measures and when an outlier from the knockout group was removed, significant decreases in aggression were found in knockout mice in some measures. Given the high variability of aggression in both groups, aggression results should be viewed as preliminary. If future work demonstrates a consistent deficit in aggression in CRFR1-/- mice, this would be consistent with the idea that a certain level of activation of CRFR1 is necessary for normal maternal defense. We previously found that icv injection of the CRF receptor antagonist, D-Phe-CRF_12–41 _(1.0 and 5.0 μg) [4] did not alter maternal aggression, but it is not known whether higher levels of antagonists would have impaired defense. One interpretation for lack of effect of receptor antagonist is that CRF neurotransmission needs to be low during lactation for the proper expression of maternal aggression, but that other modulators positively regulate levels of aggression. In other words, the loss of CRFR1 or the use of a CRF receptor antagonist might represent a floor effect whereby further lowering already low levels of CRF neurotransmission does not alter behavior [4]. Recent work suggests that acute stressors applied postpartum also decrease maternal aggression[33]. Given that central release of CRF acting on CRFR1 is an important mediator of the behavioral responses to stress, it would be interesting in future studies to examine whether or how CRFR1-/- and WT mice differ in how postpartum stressors affect maternal aggression.

We identified a difference between genotype in terms the sites of attack, with CRFR1-/- mice attacking the ventral portion of the mid-section (including belly) less frequently on the second of two test days and when both test days were averaged. Attacks to this region and also to the back/flank regions, especially in rats, has been termed offensive aggression, whereas attacks to the face/neck region have been termed defensive attacks [34]. This finding suggests that the loss of CRFR1 may alter the final output of aggression. Interestingly, in examinations of the brothers of the females tested in this study, intermale aggression was similar between genotypes, but a significant decrease in attacks by CRFR1-/- male mice to ventral portion of the mid-section (including belly) was also found [35]. Thus, for both maternal and intermale aggression, loss of CRFR1 alters the final form of aggression in the same way. Why this type of attack would be altered with loss of the CRFR1 gene is unclear, but alteration in stress reactivity or glucocorticoid levels could be involved [35]. Recent work in male hamsters found a CRFR1 antagonist, SSR125543A, administered orally lengthened latency to attack and also decreased lateral attacks [36], also suggesting a role for CRFR1 in regulating the types of attacks performed. Hence, additional studies that employ site-directed injections of CRFR1 antagonists and a careful ethological examination of aggressive sites of attack will be useful in determining whether or how CRFR1 may regulate final aggressive output.

The finding of no difference in measures of anxiety on the elevated plus maze between genotypes in lactating females on postpartum Day 6 contrasts with the previous report of decreased anxiety in CRFR1-/- mice [12]. In recent work with the WT and CRFR1-/- brothers of the dams used in this study, we replicated the finding of significantly elevated time on open arms and decreased time on closed arms in CRFR1-/- mice [35]. Because lactating females show decreased anxiety on the elevated plus maze relative to virgin females [29,37], our findings suggest that the effects of lactation remove the differences in performance between genotypes on this one test of anxiety. In a previous study on CRFR2 knockout mice, we found a lack of difference in plus maze performance between genotype in lactating mice [16] whereas differences in anxiety in virgin female mice had been reported using a different measure [38]. Thus, in two cases a loss of anxiety differences with genotype with lactation has been found. However, in rats bred for high and low anxiety, differences in anxiety persist during the postpartum period [39,40], indicating that lactation does not necessarily affect group differences in phenotype. Changes in neurotransmission during lactation of other neuromodulators that can alter indices of anxiety, such as oxytocin, prolactin, or GABA [41-43], may have helped remove or mask the normal differences in anxiety between the genotypes in this study. One possibility is that preexisting anxiety differences in CRFR1-/- and WT mice are not as great as for high and low anxiety bred rats, and these differences are more susceptible to the effects of lactation. It is also possible, though, that two days of aggression testing altered plus maze performance.

Examinations of c-Fos and Egr-1 in WT and CRFR1-/- dams allowed for possible insights into where underlying neuronal activity differences may account for maternal differences. Because these mice were untested, it is thought that immediate early gene activity seen reflects activity that is associated with the production of maternal care. c-Fos and Egr-1 were both used as indirect markers of neuronal activity because together they can provide complementary information on differences in brain activity [44-46]. Because MPOM is implicated in numerous aspects of maternal care, including nursing [1], the finding of lower Egr-1 activity in MPOM in CRFR1-/- mice suggests the possibility of subthreshold activation of this region is involved in the nursing deficit. The lower c-Fos activity in BNSTd (Egr-1 was also lower, but did not reach significance) in CRFR1-/- mice (Figs. 6A and 6B) suggests altered activity in this region could also underlie behavioral deficits. However, this portion of BNST has more frequently been associated with maternal aggression rather than other maternal behaviors [47]. Other regions approaching significant differences in activity between genotype (e.g., AAV, PIR, and PVA) could also underlie behavioral differences, but this would need to be addressed experimentally.

## Conclusion

The results from this study indicate specific nurturing deficits in CRFR1-/- mice that include nursing and licking and grooming of pups. CRFR1 is widely expressed throughout the CNS and it appears that activation of the receptor helps support the full expression of a subset of maternal behaviors. Certain other maternal behaviors were unaffected by the loss of CRFR1 (and nest building was even enhanced), so this receptor does not appear necessary for full expression of all maternal behaviors. The trend towards decreased aggression in the knockout mice coupled with high variance makes any conclusions regarding this measure preliminary. Where and how loss of CRFR1 contributes to behavioral alterations is not known, but one possible site is MPOM as was suggested by Egr-1 expression differences between genotype.

## Methods

### Animals

CRFR1-deficient male mice in an inbred C57BL/6 background [12] were produced by crossings of heterozygote CRFR1 (+/-) mice (The Jackson Laboratory, Bar Harbor, ME). Mutant males were then crossed with females (outbred hsd:ICR strain) selectively bred to exhibit high levels of maternal aggression [24]. Heterozygote CRFR1 (+/-) mice (with mixed inbred and outbred backgrounds) were then bred to produce WT and CRFR1-/- female mice used in this study. Offspring were weaned at 21 days and same sex siblings were group housed until pairing as adults. All genotyping occurred after 21 days. WT and CRFR1-/- female mice were siblings and were exposed to the same maternal and post maternal environment. For maternal behavior and aggression studies, WT and CRFR1-/- females (~50 days old) were singly housed with a breeder male (hsd:ICR strain) and following impregnation (~2 weeks), each female mouse was housed individually for the remainder of the study. Outbred breeder males were used to sire offspring because previous studies have shown that CRFR1-/- offspring of CRFR1-/- dams exhibit postnatal mortality due to improper lung development as a result of low *in utero *glucorticoid exposure [12]. Importantly, the deleterious effects of a low maternal glucocorticoid environment can be offset if the developing pup itself is heterozygote as shown in CRF-deficient mice [48]. Just prior to parturition, female mice were given precut nesting material. Polypropylene cages were changed once weekly, but after parturition, cages were not changed for the duration of the experiments. Pups were not culled, but mean number of pups between groups was almost identical. All WT and CRFR1-/- mice were given ad lib access to Harlan Tekland Mouse Breeder Diet 7004 (Harlan) and tap water. Intruder male mice (hsd:ICR strain; Harlan, Madison, WI) were sexually naïve and group-housed (4 animals/cage). Intruder males (~2 months old) were given ad lib access to regular chow. Intruder males were never used more than once per day and used for ~3 tests each. All animals were housed on a 14:10 light/dark cycle with lights on at 0600 CST. Mating success was similar between genotypes.

### Genotyping

Mice were genotyped by PCR using sense WT (5'- TCT CAG GAT TGC TAA GTT CAG-3'), sense CRFR1-/- (5'-AAC TTC CTG ACT AGG GG -3'), and a common antisense primer (5'-ACT GCT AGT GTG ATG TCC TGC -3'). Reactions were run with purified DNA from ear snips and analyzed according to vendor protocol (The Jackson Laboratory, Bar Harbor, ME).

### Maternal behavior examination

On postpartum Days 2 and 3 each dam was observed between 0900 and 1100 h. Dams were observed within their home cages and within the home housing room. Every minute for 1 hour, observers noted the maternal behaviors of the dam. This approach for examining maternal behaviors was designed in part after previous work showing that maternal performance in the morning reflects that seen at different parts of the day [49]. Those behaviors fell into one of four main categories: off nest, on nest, low arched-back nursing (LAN) (which included supine or passive nursing), or high arched-back nursing (HAN). Within each category the dam's behaviors were subcategorized into licking and grooming of pups, self grooming, eating and drinking, nest building, or no activity. The proportion of time spent in the varying activities was determined for each of the 2 days of observations and this data was used for analysis. Fig. 1 provides an overview of the timeline for all observations and testing performed.

### Maternal aggression and pup retrieval testing

On both postpartum Day 4 and Day 5 (Fig. 1), each female was exposed to an intruder male for 5 min in her home cage between 0900 and 1300 h. The pups were removed from the cage 2 min prior to the behavioral test. Removal of the pups from a dam just before an aggressive test does not diminish the expression of maternal aggression in mice [50]. The days of testing occurred within the window of peak maternal aggression that occurs from postpartum Day 4 though 10 in mice [51]. An intruder male mouse was placed in the dam's home cage and each test session was recorded on videotape and subsequently analyzed off-line to quantify maternal aggression. WT and CRFR1-/- mice were alternately tested on the same day such that the intruder mice from the same cage were used equally to test both genotypes. Thus, any previous fighting experience of intruders that may have affected outcome was evenly divided among the two groups. Maternal aggression scoring was conducted by individuals blind to experimental conditions and treatments. For quantification of maternal aggression the following features were measured: latency to first attack, number of attacks, and total duration of attacks [52,53]. Additionally, the amount of time attacking different regions of the male (including head/neck, flank/back, or the ventral portion of the mid-section, including belly) and the amount of time lunging or clawing (without physical contact) were recorded. At the completion of each test, the pups were randomly scattered in the home cage and the time to retrieve the first and fourth pup to the nest site was recorded.

### Elevated plus maze

The plus-maze apparatus was made of black Plexiglas and had two open arms (35 × 5 cm) and two enclosed arms of the same size with walls 15 cm high. The apparatus was elevated 70 cm above the ground. The arms were connected by a central square (5 × 5 cm). Indirect lighting in an otherwise dark room was adjusted to provide a standard of 6.0 lux for each test. All testing was conducted on postpartum Day 6 (Fig. 1) between 0900 and 1300 h. Mice were tested individually in 5 min sessions. Each mouse was removed from the home cage with pups remaining in the cage and was placed on the center platform facing an open arm to initiate the test session. Behaviors scored were the number of open-arm entries and the time spent in the open arm of the maze. Arm entries were defined as entry of all four paws into the arm. All test sessions were videotaped and scored by individuals blind to experimental condition.

### Data analysis

Maternal behavior and aggression variables were analyzed using a one-way ANOVA. In the cases where data were not normally distributed, then a nonparametric ANOVA on ranks (Dunn's Method or Kruskal-Wallis) was used. Analysis was conducted separately for each test day, but was also examined as a composite mean from the different test days. Although mean pup number was almost identical between groups, for further analysis of aggressive behaviors, pup number was run as a continuous variable using an analysis of covariance (ANCOVA). For one-way ANOVA analysis Sigma Stat software was used (SPSS Inc., Chicago IL) and for ANCOVA analysis Statistica software was used (StatSoft Inc., Tulsa, OK). Lunging and clawing is a mild form of aggression that does not include contact and was not included in analysis of overall levels of aggression. However, this measure was included in a separate analysis of total breakdown of different forms of agonistic behaviors within WT and CRFR1-/- mice. In the case of time to first attack, if an animal was not aggressive (no aggression shown during the test period), a time of 300 sec was assigned (the maximum possible for the test).

### Immunohistochemistry for c-Fos and Egr-1

On postpartum Day 7, half of WT and CRFR1-/- mice were untested and the other half received a maternal aggression test. Mice within a group (WT or CRFR1-/-) were randomly assigned to the no test or test condition. 100 min following the onset of the no test or test treatment, mice were briefly exposed to isoflurane anesthesia. Mice were then decapitated and the brains removed. Brains were post-fixed overnight in 5% acrolein (Sigma) in phosphate buffered saline (PBS) and cryoprotected in 30% sucrose in PBS for two days. Because no differences in aggression between genotype were identified, only untested WT and CRFR1-/- mice brains were examined (n = 5 per group). Brains were frozen on a platform and cut into 40 micron thick sections using a sliding microtome (Leica, Microsystems, Heidelberg, Germany) and stored in a cryoprotectant solution at -20 degrees C until processing for immunohistochemistry. Immunohistochemistry was run for all mice in one batch. One half of the alternate sections were processed for c-Fos and the other half were processed for Egr-1. Sections were washed in PBS in the presence of 0.2% Triton-X-100 (PBS-X), blocked in 5% normal goat serum for 1 hr, and incubated for two days at 4 degrees C with either anti-c-Fos or anti-Egr-1 antibodies. For c-Fos, tissue was first exposed to an older stock of rabbit anti-c-Fos antibodies (1:15,000, Santa Cruz, Santa Cruz, CA, catalog # sc-253) and this yielded extremely low staining. Thus, sections were rewashed and processed again using rabbit anti-c-Fos antibodies (1:15,000; Calbiochem, San Diego, CA, catalog # PC38). For Egr-1 staining, rabbit anti-Egr-1 antibodies (1:15,000; Santa Cruz, catalog # sc-189) were used. After washes in PBS-X, the sections were incubated for 90 min at room temperature in biotinylated goat anti-rabbit secondary antibodies (1:500, Vector Laboratories, Burglingame, CA), washed in PBS-X, exposed to an avidin-biotin complex (Vector) for 1 hr, washed again in PBS-X, and stained using diaminobenzidine (Sigma) as a chromagen, enhanced with 0.008% nickel chloride. The sections were then mounted, dehydrated in a series of ethyl alcohols and xylenes, and coverslipped.

### Analysis of c-Fos and Egr-1 immunoreactivity

Bright field microscopy was used for counting positive cells. The images of brain sections were projected from an Axioskop Zeiss light microscope (Zeiss, Gottingen, Germany) through an Axiocam Zeiss high resolution digital camera attached to the microscope and interfaced with a computer. Counting was based on a previously used paradigm [4,5,54]. Using boxes, cells on a specific section were automatically counted. Dimensions of boxes and brain sections used were similar to those previously used [4,5,55]. One section per brain region was used to quantify Fos or Egr-1 immunoreactivity in each animal. To ensure counting was measured consistently between samples; 1) all sections were exposed to diaminobenzidine for 10 min, 2) the backgrounds were normalized by adjusting light levels, 3) a threshold of staining levels was used to automatically distinguish between c-Fos or Egr-1 positive cells, 4) all slides were coded and the counting for each specific brain region was performed by one individual, blind to the experimental conditions, 5) only c-Fos or Egr-1 positive nuclei within a specified size range were counted, and 6) all group sections were run in one batch (that is, all c-Fos labeling was run in one batch and all Egr-1 labeling was run in one batch).

Evaluation of immunoreactivity for each marker was conducted between the two groups (WT and CRFR1-/-) using a one-way ANOVA using Sigma Stat software. In the cases where the data were not normally distributed, then a transformation of the data was used to improve normality.

## Authors' contributions

EDB and SAS genotyped mice, performed all aspects of behavioral testing, conducted the immunohistochemistry, including analysis, and contributed to experimental design. SCG, EDB, and SAS designed the experimental study. SCG drafted the manuscript. All authors read and approved the final manuscript.
